# Estimating Pilots’ Cognitive Load From Ocular Parameters Through Simulation and In-Flight Studies

**DOI:** 10.16910/jemr.12.3.3

**Published:** 2019-09-02

**Authors:** M Dilli Babu, DV JeevithaShree, Gowdham Prabhakar, Kamal Preet Singh Saluja, Abhay Pashilkar, Pradipta Biswas

**Affiliations:** a Aircraft System and Testing Establishment, Indian Air Force, Bangalore, India; b Indian Institute of Science, Bangalore, India; c National Aerospace Laboratories, Bangalore, India; d Academy of Scientific and Innovative Research (ACSIR), Ghaziabad – 201002, India

**Keywords:** Cognitive Load, eye gaze tracking, aviation safety, pupil dilation, fixation, saccades

## Abstract

Eye tracking is the process of measuring either the point of gaze (where one is looking) or the motion of an eye relative to the head. This paper investigated use of eye gaze trackers in military aviation environment to automatically estimate pilot’s cognitive load from ocular parameters. In the first study, we used a fixed base variable stability flight simulator with longitudinal tracking task and collected data from 14 military pilots. In a second study, we undertook four test flights with BAES Hawk Trainer and Jaguar aircrafts doing air to ground attack training missions and constant G level turn maneuvers up to +5G. Our study found that ocular parameters like rate of fixation is significantly different in different flying conditions. It also significantly correlated with rate of descent during air to ground dive training task, normal load factor (G) of the aircraft during constant G level turn maneuvers and pilot’s control inceptor and tracking error in simulation tasks. Results from our studies can be used for real time estimation of pilots’ cognitive load, providing suitable warnings and alerts to the pilot in cockpit and training of military pilots on cognitive load management during operational missions.

## Introduction

Aeronautical research came a long way from the days of the invention of aircraft to today’s fifth generation platforms. In present days, a fighter aircraft pilot has to undertake a plethora of tasks in addition to the primary flying task often in adverse conditions, which substantially increases pilot’s cognitive load. Pilots’ cognitive load can vary with the nature of the task undertaken, environmental effects like turbulence visibility, aircraft handling qualities, pilots mental state, training level of pilot, and so on. Physiological measures and pilot opinion rating methods exist for cognitive load measurement. The dependability and relevance of the physiological variable or pilot opinion rating is always debatable. Accurate and consistent data that quantify the handling qualities of a specific aircraft are difficult to acquire. Cooper-Harper Ratings (1) (CHR) has been used to describe and compare aircraft handling qualities for about 50 years, but they are very subjective in nature. Additionally, the data obtained through Cooper-Harper ratings are difficult to reduce (i.e. CHR cannot be averaged). Current handling qualities flight test techniques call for the test pilot to perform an operationally representative task, and then rate the aircraft using the Cooper-Harper scale (1). This rating, when pooled with other pilots’ ratings, is used to categorize the aircraft’s handling qualities. The two primary considerations of the pilot assigning a CHR are task performance and pilot workload (1). If a pilot performs as desired on the task, but is working extremely hard, then the aircraft is given a downgraded rating. Similarly, if a pilot performs poorly on the task, but is not working very hard, a downgraded rating is also given, even though the pilot might have been able to achieve better performance with a higher workload. The subjective nature and variability of how a pilot defines his or her workload may greatly influence the CHR. Once the CHRs from several different pilots have been gathered, there is no definitive guidance on interpreting the data (2).The subjective and sometimes ambiguous results obtained by qualitative handling quality ratings are inconsistent with the rest of the flight test process, where quantifiable results subjected to statistical analysis is highly desired. A high quantum of flight test effort is required for the subjective process of handling qualities evaluation.

The high sensitivity of physiological variables is required to capture fine changes in the pilot cognitive workload. Researchers have already proposed various physiological measures for estimating cognitive load such as brain related measures (ERP, EEG, MEG and brain metabolism (3) ocular measures (Blinks and pupil diameter (4,3,5)), cardiac measures, facial expressions and endodermal measures. This paper considered ocular parameters as

1. It can be measured non-invasively using Commercial Off the Shelf (COTS) sensor.
2. It can be used with flying helmet and with both clear or dark visor.
3. Existing eye tracking sensor can be used in a wide range of environmental conditions with high accuracy (0.4° of visual angle of accuracy) and sampling frequency (100 Hz).

This paper presents two studies on estimating cognitive load from ocular parameters. The first study undertook a tracking task in a fixed base simulator and compared different ocular parameters like pupil dilation, number of fixations and saccades to estimate cognitive load. We correlated ocular parameters with pilot’s control inceptor and tracking error, considered those as ground truth. The second study involved four test flights in BAES Hawk Trainer and Jaguar aircrafts performing various maneuvers between 20,000 ft to 8000ft. We collected ocular parameters from both pilot (Pilot in Command) and co-pilot (Observer Pilot) in the aircraft, using the same set of equipment used in the simulation study and compared ocular parameters during different phases of flight like take-off, cruise, maneuver and landing phases. We found the number of fixations significantly vary between pilot and co-pilot during take-off, maneuver and landing phases and also found significant correlation between number of fixation rate with rate of descent during air to ground dive training missions and with normal load factor (G) during constant G level turn maneuvers. Unique contributions of this paper are:

1. Developing a set of algorithms to estimate cognitive load from ocular parameters.
2. Designing and undertaking in flight studies with non-invasive state of the art eye gaze tracker.
3. Comparing ocular parameters during different flight phases and maneuvers and correlating those with flight parameters.

The paper is organized as follows. The next section presents literature survey on using eye gaze tracker in aviation and estimating cognitive load from physiological parameters. Section 3 and 4 describes the user studies followed by discussion and concluding remarks at sections 5 and 6.

## Related Work

Eye tracking is the process of measuring either the point of gaze (where one is looking) or the motion of an eye relative to the head. An eye tracker is a device for measuring eye positions and eye movement (6). Use of eye gaze tracking to analyze pilots’ interaction with cockpit displays dated back to 1950s (7). It can be used as a direct index of attention allocation while performing different tasks (8), to get insight into pilots’ internal (9) like situational awareness (10). Ocular parameters are also used for differentiating between novice and experienced pilots (11), testing usability of an interface (12), aircraft safety (13) and simulator training (14). De Reus et al.(15) and Biswas & JeevithaShree (16) also proposed to use eye gaze trackers as a direct controller of different displays in cockpit like Multi-Function Display (MFD) and Head Mounted Display (HMD) (17), a demonstration video can be viewed at https://cambum.net/JEMR/JEMR_Aero19.mp4. Peysakhovich et al. (18) defined a framework involving four stages of eye tracking integration in modern cockpits. They noted that at different stages of flight, eye tracking can be used for flight safety in the following ways:

- Comparison of scan paths and fixation durations to evaluate the progress of pilot trainees,
- Estimating pilots’ skills,
- Analyzing of crew’s joint attention and shared situational awareness,
- Displaying a notification at the point of pilot’s gaze to ensure its visual processing, performing an automatic maneuver and so on.

In 2015, an ATR 72-600 (GE-235) aircraft experienced a loss of control during initial climb and finally crashed into the Keelung River after take-off from Taipei Songshan Airport. A key challenge to the accident investigators was to understand why the pilot flying the aircraft misidentified the problem and shut down the wrong engine (13). To address the same, Wang et al. (13) proposed that, by recording pilots’ visual scan patterns, evidence can be obtained as a base to facilitate scientific analysis of accident investigation. As a solution, they developed a cockpit visual tracking technology which can assist accident investigations, benefit pilot training, facilitate human-centered flight deck design to prevent accidents through a more effective analysis in accident events. Hareide & Ostnes (19) investigated maritime navigation using visual scan patterns. The authors noted that with the help of maritime navigation, there can be better utilization of spatial and system awareness and consequently, situational awareness will increase safety of navigation, too. A wide variety of eye-tracking studies examined eye movements in peripheral vision displays (20), cathode-ray-tube (CRT) displays (21) and aviator helmet-mounted displays (22). In such studies, measuring eye movements can help one to understand performance in terms of task management (23) and pilots’ scanning behaviors, which in turn can be used to generate guidance for designing cockpit displays.

Wang et al. (24) used eye tracking to evaluate visibility and usability of a cockpit interface by recording gaze points, number of fixations, focusing frequency, task time and fixation order. Lin et al. (25) used eye tracking to evaluate two interface development frameworks to measure performance of pilot and eye movements. They conducted tasks involving control operation and fault detection situations. Results from eye fixation measurement showed that operator paid less attention to abstract function, fault detection tasks and state network diagram. This caused higher mental workload and reduced fault diagnosis. Similar work on fighter cockpit interface layout evaluation is reported by Wang et al. (26). They used eye tracking and identified scan-path, gaze hotspot map, number of gaze points and gaze duration by testing different interface layout schemes. They reported that different layouts have no evident effect on the number of gaze points and gaze duration, which mostly depends on type of task. Eye movements have also been used to evaluate the usability of newly developed electronic maps. Ottati et al. (27) compared eye movement patterns on different terrain features between experienced and novice pilots during a visual flight rules simulation. They reported that novice pilots devoted more visual attention outside the cockpit when compared to experienced pilots. To study electronic maps, Graeber and Andre (28) investigated how pilots visually interact with the electronic maps and suggested that training is necessary to assure proper usage of and optimal visual attention interaction with electronic moving maps. They reported that, as visibility degrades, pilots spend more time eyes-out and less time dwelling on the maps. Dill & Young’s (29) study intended to understand what pilots tend to focus their attention on. They used Research Flight Deck (RFD) platform equipped with Smart Eye Pro5 head and eye tracking for the same and collected eye tracking information regarding crew’s attention to various displays.

Eye gaze metrics can also be used to estimate pilots’ cognitive load. Studies on estimating cognitive load from ocular parameters dates back to 18th century. Charles Darwin in his book The Expression of the Emotions in Man and Animals written in 1872, indicated a correlation between widening and narrowing of eyes with emotional states. In the first decade of 19th century, Redlich (30) and Westphal (31) related pupil dilation with physical task demand, or even thinking of physical task, while Hess (32) reported change in pupil dilation with respect to viewing of photographs. In recent time, using sophisticated eye-gaze trackers, researchers found that an increase in cognitive load results in a sudden hike in pupil dilation which can be measured by a set of metrics calculated through Wavelet transform of the pupil signal considering driving simulator, aviation (5) or map reading (33) tasks. However, there is not much reported work on using COTS eye trackers in actual combat aircrafts although there is a growing need to automatically estimate pilots’ cognitive load to enhance safety of flying. Wilson et al. (3) recorded EEG and only eye blinks for air to ground attack maneuvers while in recent time (34) reported results on gaze controlled interface in an Avro HL 768 aircraft. Henk et al. (35), investigated eye gaze patterns to assess situational awareness during scenarios of instrument malfunctions. They measured eye gaze fixation rates, dwell time and visual scanning entropy and found that these measures add more insights into the situation than only subjective self-rating metrics. Ratwani et al. (36) conducted a similar analysis on an UAV (Unmanned Aerial Vehicle) task and concluded that visual attention allocation and visual scanning are key components to operators’ situational awareness. Prabhakar and Biswas (37) estimated cognitive load from ocular parameters in automotive environment and ocular parameters significantly correlate with parameters measured from T7 electrode of EEG signal. Siegenthaler et al. (38) found decrease in microsaccade rate with increase in task difficulty. Their study of arithmetic task involved increasing load on working memory. Gao et al. (39) reported suppression of microsaccade rate with respect to increase in arithmetic task difficulty for non-visual cognitive processing. Dalmaso et al. (40) reported that microsaccade rate drops with high demand task. Krejtz et al. (41) captured pupil diameter and microsaccades as indicators of cognitive load. He reported a mild evidence of decrease in microsaccade rate with increase in difficulty of task. He also reported a strong evidence of increase in magnitude of microsaccade with increase in difficulty of task. However, these studies used a chin rest to arrest head movement which limits the application of such technology to be used in real world systems. Tokuda et al. (42) estimated mental workload from saccadic intrusion (SI) using a driving simulator based study. He reported a strong evidence of increase in velocity of SI with increase in difficulty of task.

In summary, most researches on eye tracking defined a set of Areas of Interests (AoI) and analyze saccadic gaze movements between and within those areas of interest. Such analysis has already been conducted for different fixed and rotary wing aircraft as well as fighter, civilian and transport aircrafts. A variety of variables like duration and count of fixations, length of saccades and so on have been analyzed for investigating pilots’ attention and lack of attention as well. Studies estimating cognitive load mainly considered different parameters of eye blink and pupil dilation (4). However, most studies used fixed position eye gaze trackers while the following study used a head mounted tracker supporting more head movement than screen mounted trackers. In the following sections, we reported two studies – one involving a flight simulator and another using combat aircrafts reporting a set of ocular parameters for different military aviation tasks undertaken by military pilots.

## Study 1 – Flight Simulator Study

The experimental piloting task was designed to effect fine variation in cognitive load, instead of gross variation. The pilot pitch tracking task was superimposed with constantly closing boundary to bring in gradual increase in cognitive load in fine steps (43). In the following subsections, we have described our study in detail.

**Participants:** We collected data from 14 participants of the ranks ranging from Squadron Leader to Group Captain of Indian Air Force, average age 35.43 years (stdev 4.27 years), average flying experience 13.29 years (stdev 5.99 years) and 1766.43 hours (stdev 752 hours).

**Figure 1:**
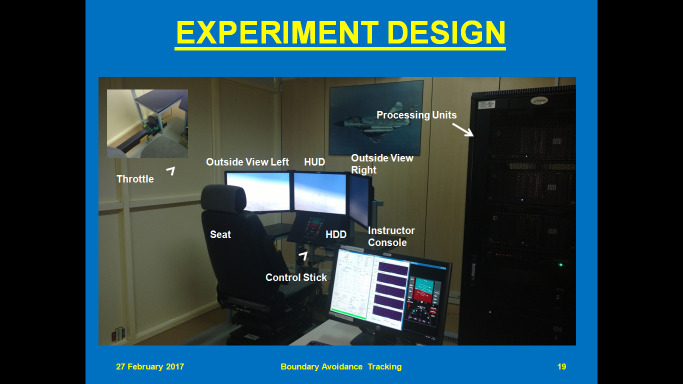
Experimental Set Up for Cognitive Load Estimation

**Material:** We used a fixed base fixed wing variable stability simulator. It consists of five Central Processing Units (CPUs), three 24’’ monitors for outside view, one 22’’ monitor for instructor console, one 18.5’’ monitor for Head Down Display (HDD), one control stick, two throttles, two rudder paddles and one seat. The simulator is based on a distributed architecture where five different processors handles five different functions and they are interlinked to provide a flight simulation. The simulator architecture consists of aircraft model, out of window visuals, avionics displays, data analysis tools and an instructor station that works. The instructor station controls all program execution. Figure 1 shows different components of the simulator. Experiments are undertaken using Lear Jet Aircraft Model. Eye gaze was recorded using a commercial wearable eye gaze tracker (44).

**Figure 2:**
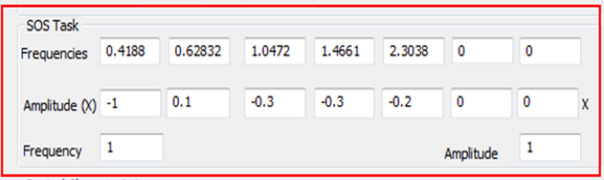

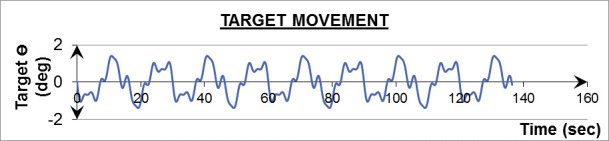
Tracking Task (HUD View)

**Figure 3:**
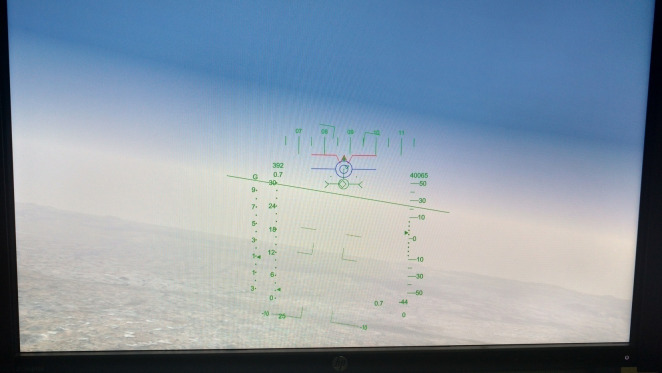
Sum of Sine (SOS) Task

**Figure 4:**
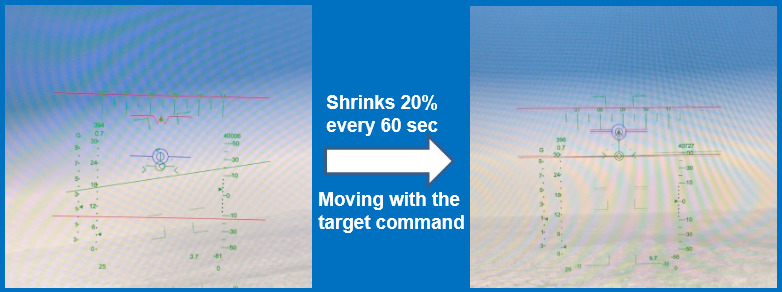
Two Opposing and Constantly Closing Boundaries

**Figure 5:**
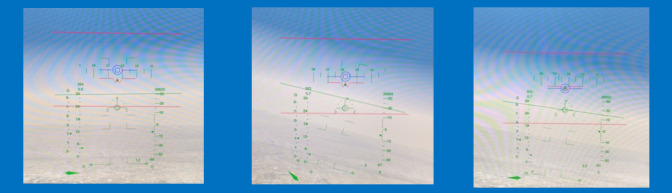
Secondary Arrow Task at Left Bottom

**Design:** The experiment was focused on a task involving longitudinal tracking of target. Gray (45) hypothesized that the boundary avoidance tracking theory which considers the pilot tracks the boundaries imposed in flying when they become dominant in terms of threatening conditions. In the frame of a preliminary simulator study with five participants, the flight test technique which is associated with boundary avoidance tracking was performed by Niewind (43), and the concept was investigated in detail (46). Niewind’s study confirmed that the new technique can systematically and gradually raise the pilot gains in a buildup fashion. As shown in figure 2, the blue colored symbol (blue concentric circles with horizontal line) is the target and the red “W” symbol is the own aircraft symbol (center tip of W indicating the nose of aircraft). Red colored boundaries are equidistant from the target's present position and moves in synchronization with the target movement. The boundaries remain in a particular state for 60 seconds and then shrink by 20% after every 60 second. For example, at time equals to zero seconds, boundaries are at +5° and -5° with respect to the target, then it would reduce to +4° and -4° after 60 seconds and so on. Figure 4 shows the shrinking phenomenon of boundaries. The optimized value of sum of sines suitable for the experiment was used to provide adequate challenge in predictability in the pitch tracking task. The sum of sines signal of the tracking task was defined as shown in figure 3 below. The frequency and amplitude defined for the five sine waves are shown in figure 3a. Thus, the target moved in pitch as per the resultant signal of sum of the five sine wave signals. The resultant signal for tracking task defined is as shown in figure 3b. The design of the task ensured change in pilot’s cognitive load by two horizontal lines constantly closing in together and acting as boundaries. The two opposing and constantly closing boundaries were designed as shown in figure 4. Pilots were briefed not to hit the boundaries while undertaking pitch tracking task. A secondary arrow task (figure 5) was designed as shown in figure 5 to further increase the cognitive load (46). The task was the pilots to depress a button in throttle as per the clockwork position of the arrow shown at left top of the display.

**Results**: We analyzed data for the following three conditions:

- C1: Flying without any boundaries or operating any secondary tasks
- C2: Flying within boundaries
- C3: Flying within boundaries and perform the secondary task

We selected the following set of ocular parameters based on previous research.

1. **Pupil dilation:** Previous research noted a hike in pupil dilation for change in affective state. We used signal processing algorithms to detect sudden hikes in pupil dilation.
2. **Saccadic intrusion:** Saccadic intrusions (47) are conjugate, horizontal saccadic movements which tend to be three to four times larger than the physiological microsaccades and take the form of an initial fast eye movement away from the desired eye position, followed, after a variable duration, by either a return saccade or a drift (47). It is characterized by a type of eye gaze movement where eye gaze returned to same position between 60 and 870 milliseconds interval and maximum deviation of eye gaze within the interval is more than 0.4° in X-axis.
3. **Number of eye gaze fixations and saccades:** There are different algorithms to extract fixations and saccades from raw eye gaze coordinates depending on spatial distribution of gaze points (48), bearing angle and velocity of eye gaze movement (49,50). In this particular study, we used a velocity-based threshold to detect saccades and fixations from raw gaze point.

We did not include eye blinks as Wilson et al. (3) already reported results on eye blink for similar study and COTS eye trackers do not detect and automatically distinguish between exogenous and endogenous eye blinks. It can only tell when eye gaze is not detected, and it is difficult to decide whether eye gaze is not detected due to vibration in the cockpit or eye blink. We analyzed pupil dilation using short term Fourier Transform and measured the number of fixations, saccades and number of occurrences saccadic intrusion, detailed algorithms are furnished below.

**Measure of Pupillary Cognition (MPC):** An FFT (Fast Fourier Transform) was performed over the raw data of pupil dilation (37). We added the magnitude values of bins corresponding to 1Hz to 5Hz (51) in the single-sided spectrum. We did this procedure for full length of the signal as well as in time buffers of 1 second for real-time implementation. We calculated the *MPC* for each second and store in an array *MPC*_*C*1_ corresponding to *data*_*C*1_. We repeated the same procedure to calculate *MPC*_*C*2_ and *MPC*_*C*3_ from *data*_*C*2_ and *data*_*C*3_ respectively. For each participant, we calculated the mean of *MPC*_*C*1_, *MPC*_*C*2_ and *MPC*_*C*3_ and checked if *MPC*_*C*3_ > *MPC*_*C*2_ and *MPC*_*C*3_ > *MPC*_*C*1_. We repeated for all participants and checked if *MPC*_*C*3_ was significantly greater than *MPC*_*C*2_ and *MPC*_*C*1_.

**Saccadic Intrusions (SI).** We extracted 2D gaze positions (x,y) and their corresponding timestamps from the data file of Tobii glasses and stored in x, y and t respectively. The camera resolution of Tobii glasses is 1920×1080 pixels and the horizontal visual angle is 160° (44), so the number of pixels within 0.4° is 4.8 pixels.

1 0.4°/160°×1920=4.8 pixels

We used the algorithm described in Biswas & Langdon (52) to detect saccadic intrusion from raw gaze stream.

**Number of saccades and fixations** were extracted using the IV-T fixation filtering algorithm (50) of Tobii Studio software with velocity-based threshold set at 30°/sec.

**Figure 6:**
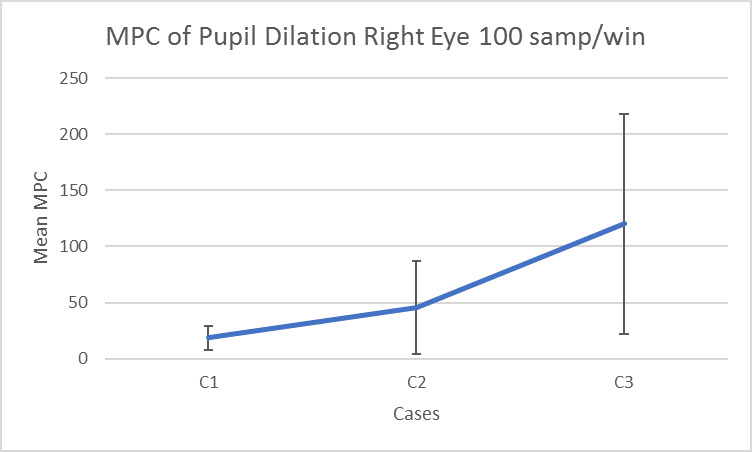

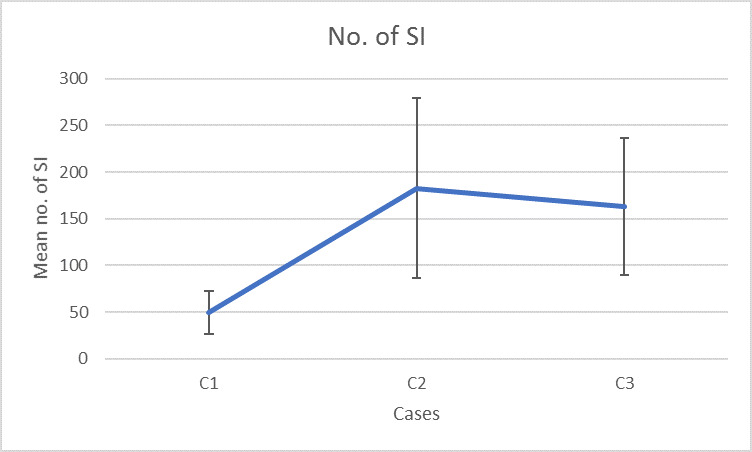

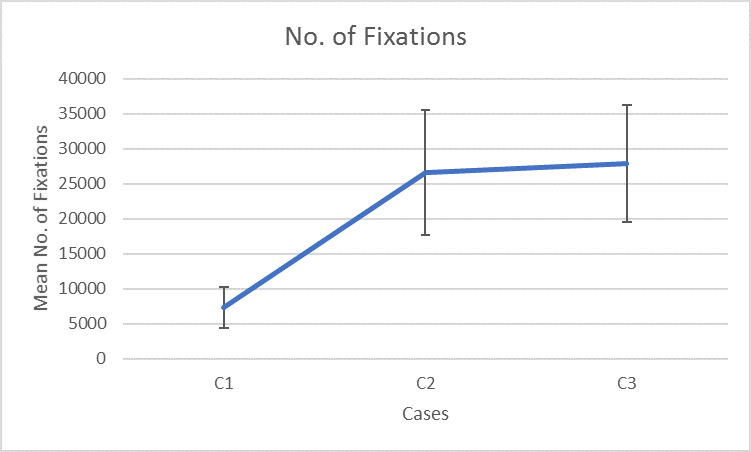

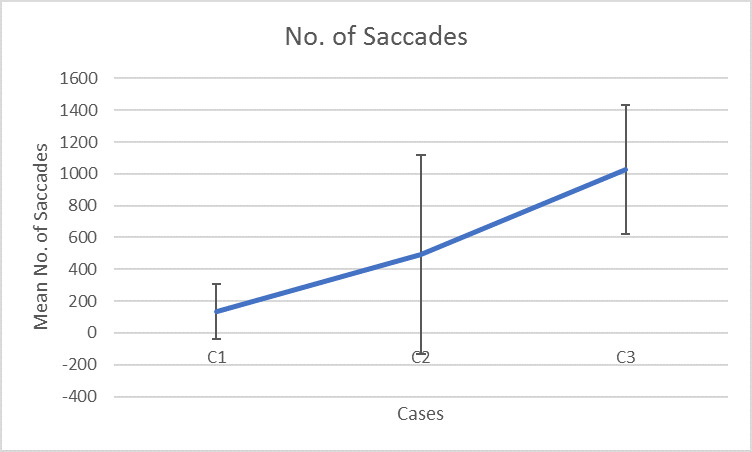
Ocular parameters for different conditions

**Figure 7:**
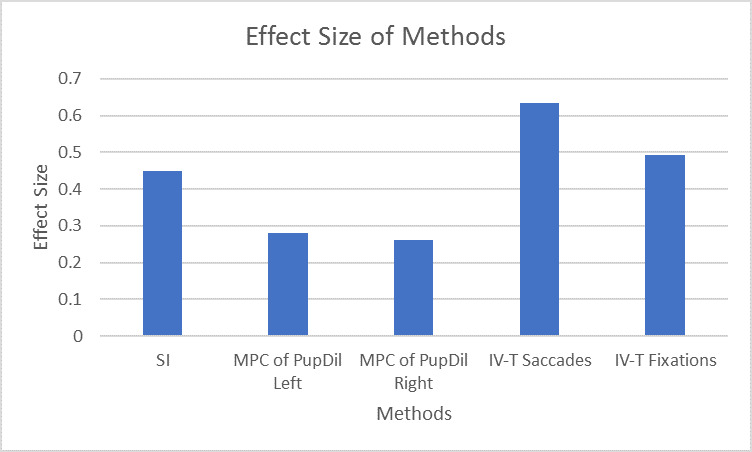
Comparing different ocular parameters for estimating cognitive load

We undertook one-way ANOVA and all parameters were significantly different for the three conditions at p<0.05 (Figure 6). We have calculated the effect size in terms of β^2^ and figure 7 below is showing the effect size for different parameters.

- Pupil Dilation Left Eye [F (2,36) =7.18, p < 0.05, β^2^ =0.28], pairwise t-test is significant at p<
- Pupil Dilation Right Eye [F (2,36) =8.67, p < 0.05, β^2^ =0.26], pairwise t-test is significant at p<0.5 between all pairs
- Number of Saccadic Intrusion, [F (2,36) =12.28, p < 0.05, β^2^ = 0.45], pairwise t-test is significant at p<0.5 between C1, C2 and C1, C3
- Number of Saccades [F (2,36) =12.51, p < 0.01, β^2^ =0.63], pairwise t-test is significant at p<0.5 between C2, C3 and C1, C3
- Number of Fixations [F (2,36) =30.06, p < 0.01, β^2^ =0.49], pairwise t-test is significant at p<0.5 between C1, C2 and C1, C3

The number of saccades and fixations was found to have highest effect size. We undertook a set of pairwise t-tests for all dependent variables and found the following differences as significant:

- All ocular parameters are significantly different at p<0.05 between conditions 1,2 and 1, 3.
- Pupil dilation and Saccades are significantly different at p<0.05 among all pairs of conditions.

To analyze the fine variation in cognitive load we analyzed the variations in number of saccades and fixations between each boundary size to observe the effect caused by boundaries on cognitive load (43). We analyzed the correlation of number of saccades and fixations with pilots’ control inceptor movement and tracking error in further details. As discussed earlier, conditions 2 and 3 required pilots to fly within an envelope and the boundary of envelope kept on changing every 1 minute. We divided the whole data stream for each individual boundary values and each boundary value has 60 secs duration. We calculated the number of saccades and fixations within each boundary duration for conditions 2 and 3 and correlated them with the following three parameters:

- **Duty Cycle** is a measure of Pilot Inceptor Workload (PIW) and is measured by the percentage of time with significant stick movement (43).
- **Aggressiveness** is another measure of pilot inceptor workload and measured by the RMS value in deg/sec of the speed with which the stick is moved (43).
- **Error in Performance** is the difference at any point in time between the pitch angle of the test aircraft and the target aircraft and measured in unit of degree. It is a measure of task performance through the tracking error.

**Figure 8:**
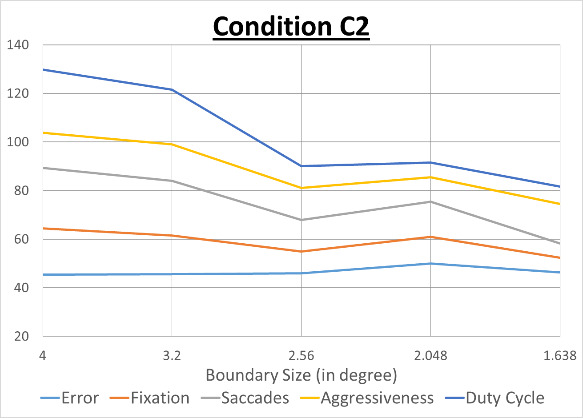

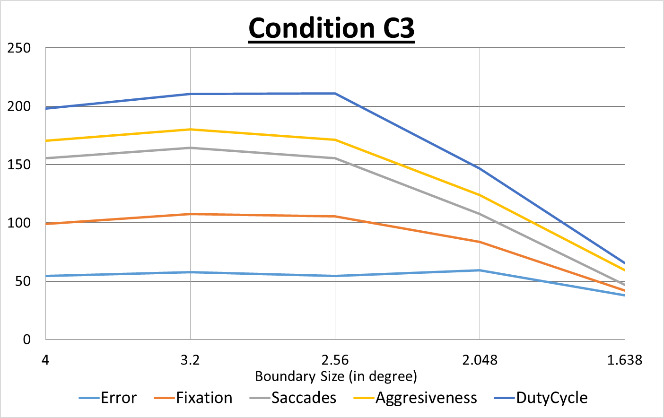
Change in parameters with decreasing boundary sizes

Figure 8 below plots each boundary duration on x axis and median values for all parameters on y-axis. For plotting all variables in a single graph, we multiplied values of error and aggressiveness by 10.

It may be noted that all curves follow similar patterns across different boundary values. We have found highest values of correlation between number of fixations and error in performance (0.92 for case 2 and 0.96 for case 3).

**Discussion:** The experimental task was designed to increase the difficulty level of the flying tasks from conditions C1 to C3. Further, to capture fine variations in pilots’ cognitive load, the task envelope in terms of boundary limits were decreased in fine steps every 60 seconds, within C2 and C3 conditions. The study demonstrates that ocular parameters like hike in pupil dilation, saccadic intrusion and number of saccades and fixations vary significantly with change in pilots’ cognitive load. In conditions C2 and C3, as the boundary values of the tracking task were gradually decreased, and it was expected that closing boundaries compel the pilots to change their control strategy to remain within the closing boundaries, which result in fine variations in pilots’ cognitive load between each boundary size. The influence of closing boundaries on pilot control strategies is inferred from marginal variation in average error and significant variation in PIW. Number of saccades and fixations are found to correlate with variations of PIW in both C2 and C3 conditions. We may infer that by recording ocular parameters, it would be possible to predict variations in pilot’s cognitive load. By continuously monitoring ocular parameters, it would be possible in future to constantly monitor pilots’ cognitive sate and early intervention in case of safety critical increase in stress level. However, accuracy of these ocular parameters may change under variable lighting and vibrating conditions inside an actual aircraft. The following study recorded the same set of ocular parameters inside combat aircrafts undertaking representative military exercises.

## Study 2 - In-Flight Analysis using Hawk Aircraft

The previous study shows that ocular parameters can estimate pilots’ workload and in particular, the number of fixations highly correlate with pilot’s control inceptor and tracking error showing pilots’ flying strategy under adverse condition. The subsequent question was, can similar results be obtained in a real flight and whether the eye tracking device will work under high G forces and record ocular parameters. The following sections describe a study undertaken in BAES Hawk and Jaguar aircrafts undergoing high G maneuvers and air to ground dive attacks training missions. We compared ocular parameters of pilot and co-pilot during different phases of flights and maneuvers.

**Study Description:** The BAE Systems Hawk is a British single-engine, jet-powered, twin seater trainer aircraft in tandem seating configuration. Three test flights were undertaken to evaluate the utilization of eye tracker for inflight estimation of cognitive load of military pilots while undertaking routine training operational tasks. A fourth study was undertaken using a Jaguar aircraft, which is used for close air-support and ground attack missions. Details of the crew is furnished in table 1.

**Table 1:** Details on Crew

Seat	Age (in Years)	Responsibility	Flying experience	Aircraft flying background
Pilot1	35	Pilot in command	1920 hrs	Advanced Multirole Aircraft
Pilot2	35	Pilot in command	2100 hrs	Bomber Aircraft
Co-Pilot	38	Observer pilot	340 hrs	Flight Test Engineer

The first three flights were flown by the same crew so that physical related differences and experience related differences in ocular parameters between pilots will not affect the results. Further the first three flights were undertaken within two days and at same geographical location thereby ambient lighting and prevailing atmospheric conditions were ensured similar in the first three flights. Further test flights were undertaken in calm wind conditions and visual meteorological conditions (VMC). Hence during inflight tests differences in ocular parameters were ensured minimal to negligible by careful design and planning of flights. The fourth flight used a different aircraft (Jaguar) and crew (pilot2) undertaking air to ground attack training tasks. The profile of the flights is described in Table 2 below.

**Table 2:** Flight Profiles

Sl No	Objective	Profile
**Flight #1**	Maneuvering flight with head mounted eye tracker on Observer pilot	Take-off – climb – level flight to Local Flying Area – Constant G (3G and 5G) level turns both sides each – Vertical loop – Barrel Roll – Air to Ground dive attack training missions – Descent – ILS Approach and landing
**Flight #2**	Maneuvering flight with head mounted eye tracker on Pilot in Command	Take-off – climb – level flight to Local Flying Area – Constant G (3G and 5G) level turns both sides each – Vertical loop – Barrel Roll – Air to Ground dive attack training missions – Descent – ILS Approach and landing
**Flight #3**	Non - Maneuvering flight with head mounted eye tracker on Pilot in Command	Take-off – climb – level flight to Local Flying Area – Straight and Level cruise with gentle level turns – Descent – ILS Approach and landing
**Flight #4**	Maneuvering flight with head mounted eye tracker on Pilot in Command	Take-off – climb – level flight to Local Flying Area –Air to Ground dive attack training missions – Descent – ILS Approach and landing

**Figure 9:**
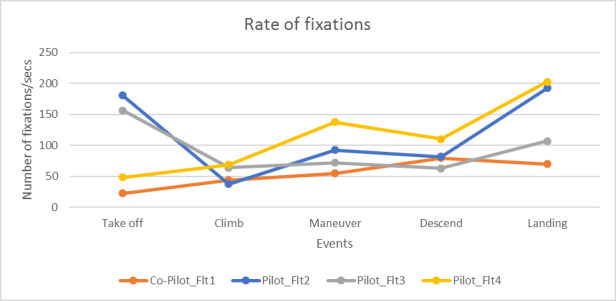
Variations in fixation rate

**Results:** Initially, we annotated the whole flight duration into different phases with the help of the pilot and co-pilot. A sample dataset can be downloaded from https://tinyurl.com/y5yc36lp. We also took help from video recorded by the eye gaze tracker. For each phase, we calculated all ocular parameters using the same algorithms used in previous study and divided it by the duration of the phase. Figure 9 below shows the variations in the rate of fixations between pilot and co-pilot in different phases of flight.

In a previous study, Di Norcera et al. (53) reported higher cognitive load of pilots during take-off, maneuver and landing phases of flights through NASA TLX scoring. It may be noted from figure 9 that the rate of fixations was more than twice for the pilot than the co-pilot during take-off, maneuver and landing. The fixation rate for the third flight follow similar pattern as the second flight, it was higher during take-off and landing than climb and descend phases. For the fourth flight, the rate of fixation was lower during take-off but increased during maneuver and landing from the cruise and descend phases.

**Figure 10:**
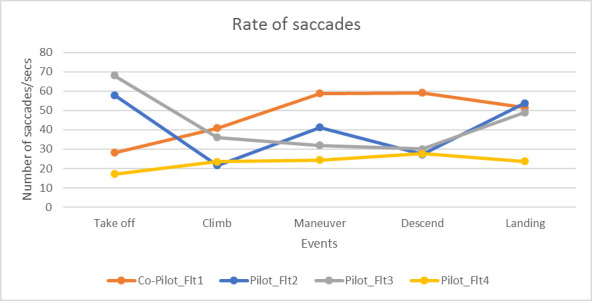
Variations in saccade rate

For pilot, the rate of saccades follows similar pattern as the fixation rate for both flight 2 and 3. However, for co-pilot, the rate of saccades was highest during maneuver and lowest during take-off (Figure 10).

**Figure 11:**
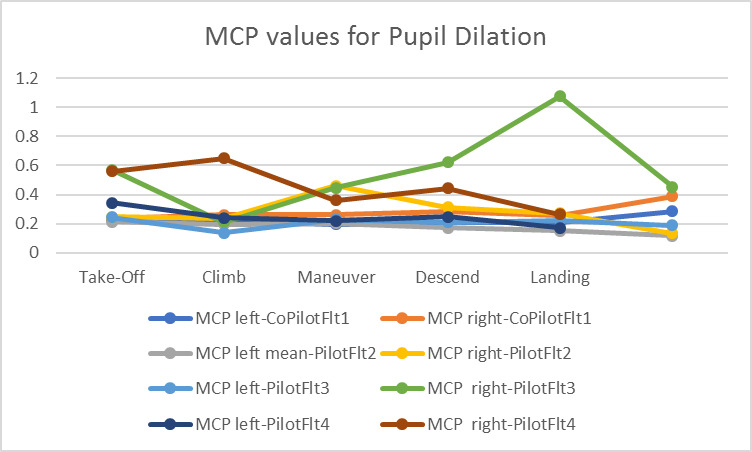

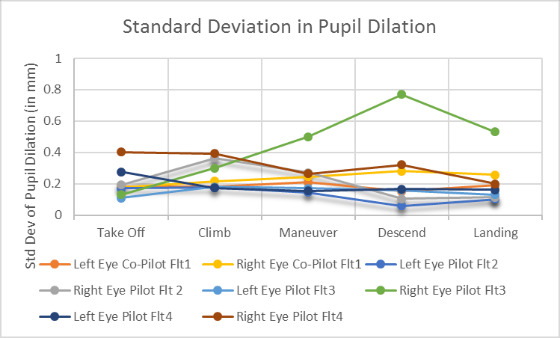
Variations in pupil dilation

We calculated the MPC values of pupil dilation as the simulator study and figure 11 below shows the variations across the three flights. We also measured the standard deviation of pupil dilation during different phases of flight. It was highest (0.77 mm) during the descend phase of flight 3, otherwise between 0.1 and 0.35 mm for all other cases.

**Figure 12:**
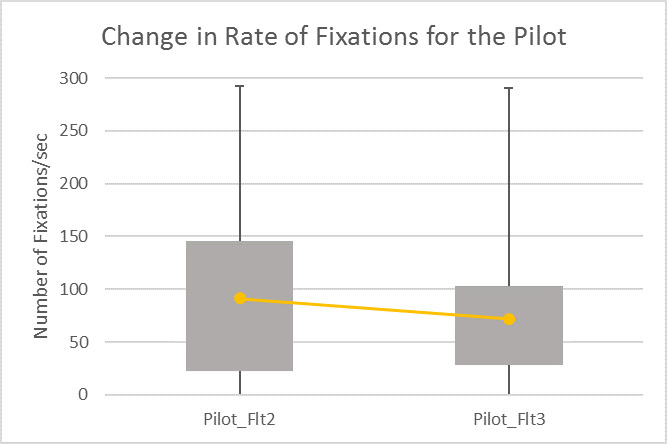
Comparing average fixation rates

As the fixation rate was higher for pilot during take-off, landing and maneuver phases, we analyzed it in further details. We calculated the number of fixations in every 5 secs interval for flights 2 and 3. The fixation rate was significantly higher for flight 2 than flight 3 for the same pilot (Figure 12) in an unequal variance t-test [t(0,579) = 4.25, p<0.001]. The variance was also higher in flight 2 than flight 3 (Figure 12).

**Figure 13:**
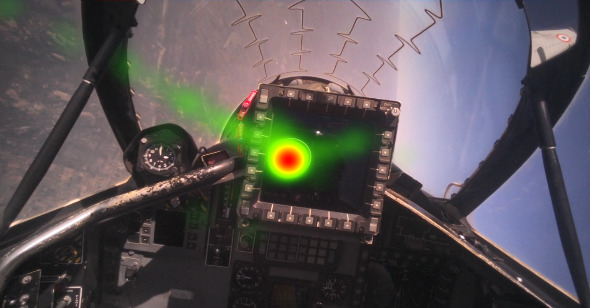

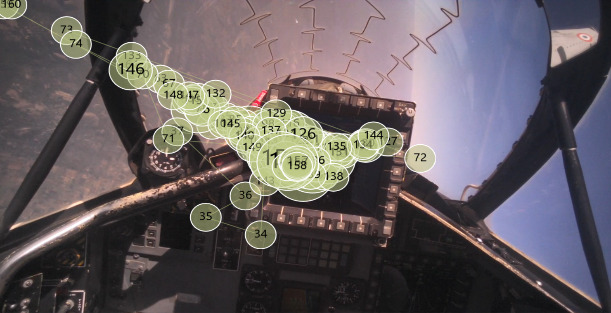
Eye gaze fixations during constant G level maneuver

**Figure 14:**
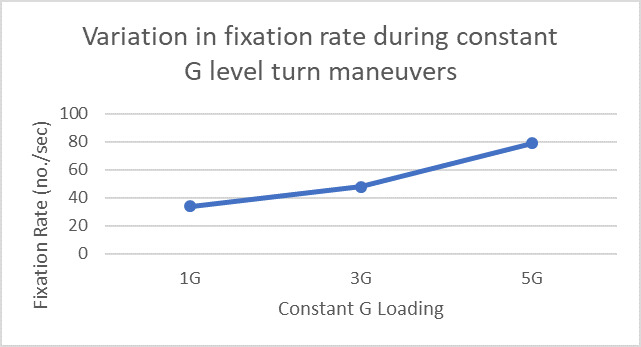
Variations in fixation rate during constant G level turn maneuvers

We further analyzed fixation rate for different maneuvers of flight 2. during constant G level turn manoeuvers. Figure 13 shows the heatmap and sequence of eye gaze fixations. The fixation rate monotonically increased with increase in G values for the different values of constant G level turn manoeuvers (figure 14).

**Figure 15:**
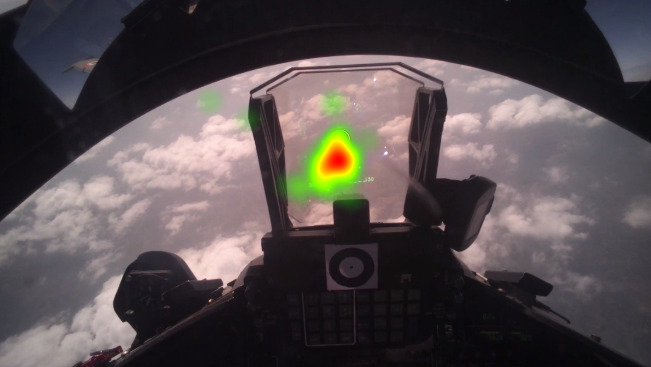

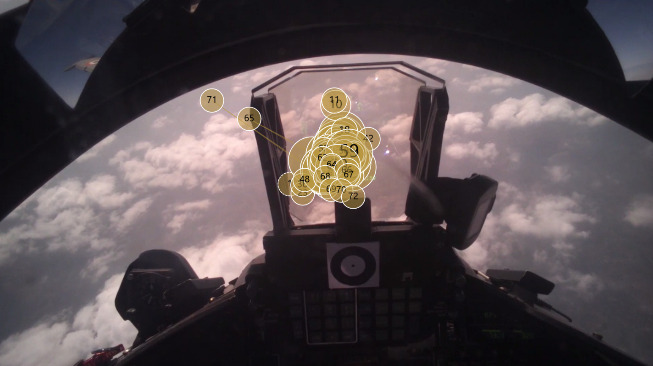
Eye gaze fixations during 1st Air to Ground attack maneuver

After the high G maneuvers, we analyzed the fixation rate for seven air to ground dives. Figure 15 plots the heatmap and sequences of eye gaze fixations for the first dive. It may be noted that the pilot fixated attention at the middle of HUD (Figure 15) and saccadic gaze movements were limited to a small region of the visual field.

**Figure 16:**
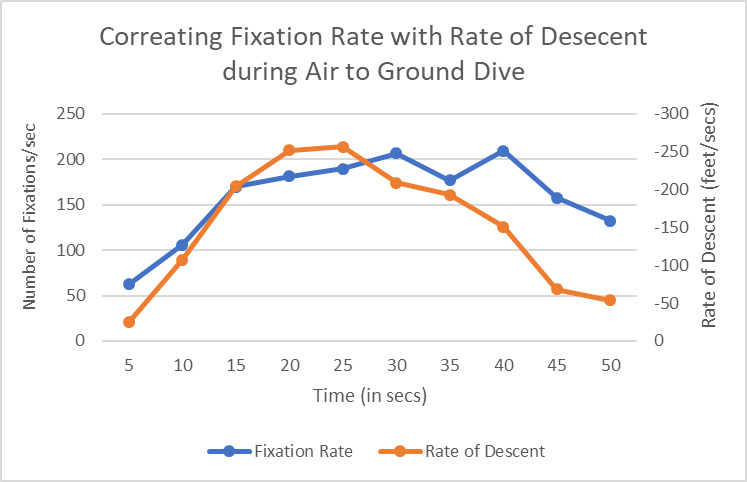
Fixation Rate varies according to the Rate of descent

We plotted the fixation rates at a 5 secs interval for seven air to ground dive attack training missions and noted that the fixation rates increased during the first half of the dive and the same decreased during the last half of the dive. The values of fixation rates were higher than the average fixation rate (indicated by the dotted line) during the whole maneuver. Next, we calculated the change in altitude (rate of descent) and related it with fixation rate. It may be noted that the onboard sensors in the flight recorded altitude and only the magnitude of the vertical velocity while the rate of descent has both magnitude and sign - a negative rate of descent means the plane was descending downward while a positive value indicates a climb. Figure 16 plots the average fixation rate and rate of descent over time for all seven air to ground attack dives. The figure shows both fixation rate and rate of descent had similar shape (inverted U), means while the vertical speed increased during first half of the dive, fixation rate also increased and as vertical speed decreased during second half of the dive, fixation rate also decreased. The fixation rate statistically significantly correlated with rate of descent for all seven air to ground dives (r>0.7, p<0.05).

**Discussion:** In study 1, we collected data from 14 pilots and flight test engineers in a laboratory study and found ocular parameters are significantly correlated with pilots’ workload. This study aims to externally validate our result in variable lighting and vibrating conditions of an actual aircraft. We found that the fixation rate measured based on a velocity based algorithm statistically significantly correlate with rate of descent and constant load factor of aircraft. Earlier studies either reported ocular parameters in different flight phases (53) or eye blink rate during air to ground dive attacks (3). We analyzed fixation and saccade rates and pupil dilation using standard statistical hypothesis testing procedures and reported results on flight phases, air to ground and constant g load maneuvers. A sample data from one flight can be downloaded from the following link for any further analysis https://tinyurl.com/y5yc36lp .

## Overall Discussion

This paper presented two studies on comparing ocular parameters for different flying conditions with respect to combat aircrafts. The first study was conducted in a high-end simulator. We found ocular parameters, in particular number of saccades and fixations significantly increases with pilots’ workload. We used pilot’s control inceptor and tracking error like duty cycle and aggressiveness as ground truth and number of fixations statistically significantly correlated with the ground truth metric. The second study involved three flights in Hawk and Jaguar aircrafts. The aircraft went through high G and air to ground attack maneuvers. The fixation rate was higher in take-off, landing and maneuvering stages of flight and also increased during air to ground dives and high G maneuvers. In a previous study, Di Norcera et al. (53) reported more variations in fixation rate at the take-off and landing phases of flight compared to cruise phase and reported higher cognitive load during take-off and landing through NASA-TLX scores as well. The rate of fixations was significantly higher for the flight undertaken high G and air to ground maneuvers (flight 2) than the one only undertook level flight (flight 3). It may be noted from the heat maps of eye gaze fixations that saccadic gaze movements were limited to small region of visual field and higher number of fixations cannot be attributed to visual search. The eye gaze was fixated to the ground target during the air to ground dives and the rate of fixations was directly proportional to the rate of descent. As the pilot was approaching target, his vertical speed was increasing and similarly number of fixations increased and while he was easing out of the dive, the rate of fixations decreased.

The pupil dilation-based measurements were significantly different for the simulation study but not for the in-flight study. Our metrics (MPC and standard deviation) measures changes in pupil dilation and as during the maneuvers the aircraft cockpit was under variable lighting condition, the pupil dilation was affected not only by the pilots’ workload but also by the change in external sunlight exposure.

**Ground Truth:** Cognitive load estimation studies generally use a ground truth metric and new parameters are correlated with ground truth. Unlike previous studies (54) we did not use another physiological metric as ground truth, rather used the flying conditions as baseline and experimental conditions. Earlier studies have already correlated ocular parameters with physiological parameters like EEG (55), EKG, EMG (56) for reading tasks and driving related tasks in automotive environment (37). In the simulation study, we used the flight pilot’s control inceptor and tracking error as ground truth. In the in-flight study, we chose the maneuvers which are already found by previous work to increase pilots’ workload (3). Hence, we correlated ocular parameters like fixation rate with flight parameters like rate of descent during air to ground dives and normal load factor (G) of the aircraft. Our study shows that existing COTS eye gaze trackers can be used to measure ocular parameters in high G environment and by measuring ocular parameters, in particular fixation rate, we can estimate pilots’ workload.

**Placement of Eye Tracker:** We investigated the issue of placing the eye tracker in the cockpit of various existing aircrafts of the Indian Air Force. For a screen mounted tracker, it can track eye gaze of pilots if it is placed below the HUD of the front cockpit or below the topmost MFD of the rear cockpit for BAES Hawk Trainer and Sukhoi 30 MKI platforms. The screen mounted eye tracker has the advantage of being completely non-invasive and it can track eyes even through the dark visor (16). However, a screen mounted eye tracker will lose tracking if the pilot is looking sideways. A helmet mounted eye tracker will be helpful for tracking eyes for any head position, but it will require more stringent airworthiness testing than a screen mounted tracker as it will be part of ejection system. Our present research is investigating controlling a HMDS through a helmet mounted eye tracker along with estimating cognitive load.

**Value Addition:** Earlier work investigated ocular parameters for cognitive load estimation in laboratory-based trials (57) and also measured only eye blinks in in-flight study. There is not much reported work on measuring ocular parameters in variable G environments. Adelstein et al. (58) and colleagues reported “significant degradations in both error rate and response time in a reading task at 0.5 and 0.7 g for 10-pt, and at 0.7 g for 14-pt font displays”. However, we did not find any study that used COTS eye tracking device at high G environment up to 5G and investigated different ocular parameters for realistic combat missions. Our results can easily be used for training pilots in terms of cognitive load management during highly demanding missions in terms of cognitive load and situational awareness. Results from our studies can also be exploited for real time estimation of pilots’ cognitive load and providing suitable warnings and alerts to the pilot in the cockpit. In summary, the main findings from our study are

1. Commercial eye gaze tracking glasses can be used to measure ocular parameters in combat aircraft up to +6G.
2. Rate of fixation measured using the algorithm discussed (49,50) increases for tasks demanding higher cognitive workload from pilots.
3. All pupil dilation based metrics should be tested in variable lighting and vibrating conditions of an actual aircraft before using those for cognitive load estimation.

A presentation on use of eye gaze trackers for military aviation can be found at https://youtu.be/y7S8U3QA3do

**Limitations and Future Work:** In this study, we reported values of a set of ocular parameters at various levels of workload of pilots’ in a simulation study and also inside a combat aircraft. We did not use a second physiological metric of ground truth. We are planning to involve an EEG tracker like Prabhakar& Biswas’s (37) study in a future experiment with flight simulator. Similarly, we are investigating availability of an alternative device that can be carried in parallel to an eye gaze tracker inside the helmet of a pilot and can be used as an additional ground truth metric during different maneuvers.

## Conclusion

Estimating pilots’ cognitive load is a well investigated problem although still now there is no real time estimation system deployed on any combat aircraft. There is plethora of studies available relating ocular parameters to cognitive load, with a few studies in military aviation environment as well. We used a COTS sensor to record ocular parameters like fixations, saccades and pupil dilation. We reported two studies – one involving a fixed base variable stability flight simulator and another involving three flights in BAES Hawk and Jaguar aircrafts maneuvering in high G conditions and undertaking various training combat missions. Both studies found significant correlation between pilot’s cognitive load and ocular parameters, in particular, rate of fixations. Our study confirmed that ocular parameters can be detected using COTS sensor in military aviation environment under high G conditions and can be used to estimate pilots’ cognitive load in real time.
